# Prostate-specific membrane antigen PET imaging and immunohistochemistry in adenoid cystic carcinoma-﻿a preliminary analysis

**DOI:** 10.1007/s00259-017-3737-x

**Published:** 2017-06-07

**Authors:** Thomas J. W. Klein Nulent, Robert J. J. van Es, Gerard C. Krijger, Remco de Bree, Stefan M. Willems, Bart de Keizer

**Affiliations:** 1 Department of Head and Neck Surgical Oncology, Utrecht Cancer Center, University Medical Center Utrecht, P.O. Box 85500, Heidelberglaan 100, Utrecht, 3508 GA The Netherlands; 2 Department of Oral and Maxillofacial Surgery, University Medical Center Utrecht, Utrecht, The Netherlands; 3 Department of Radiology and Nuclear Medicine, University Medical Center Utrecht, Utrecht, The Netherlands; 4 Department of Pathology, University Medical Center Utrecht, Utrecht, The Netherlands

**Keywords:** PSMA, Adenoid cystic carcinoma, AdCC, PET/CT, Immunohistochemistry, Head and neck cancer, Salivary gland

## Abstract

**Background:**

Adenoid cystic carcinoma (AdCC) of the head and neck is an uncommon malignant epithelial tumour of the secretory glands. Many patients develop slowly growing local recurrence and/or distant metastasis, for which treatment options are limited. A retrospective analysis of 9 AdCC patients was conducted to analyse the visualization of AdCC on PSMA PET/CT and to investigate the expression of PSMA on primary, recurrent and metastatic AdCC tumour tissue using immunohistochemistry.

**Results:**

Local recurrence occurred in six patients and eight developed distant metastasis. All PET/CTs depicted PSMA-ligand uptake. Four PSMA PET/CTs showed suspected residual disease, eight scans depicted uptake in areas suspected of distant metastasis. Median Maximum Standardized Uptake Value (SUV_max_) in local recurrent and distant metastatic AdCC was 2.52 (IQR 2.41–5.95) and 4.01 (IQR 2.66–8.71), respectively. All primary tumours showed PSMA expression on immunohistochemistry (5–90% expression), as well as all available specimens of local recurrence and distant metastases.

**Conclusion:**

PSMA PET/CT is able to detect and visualize local recurrent and distant metastatic AdCC. PSMA-specific targeting is supported by PSMA expression on immunohistochemistry.

## Background

Adenoid cystic carcinoma (AdCC) is an uncommon malignant epithelial tumour of the secretory glands in the head and neck region, accounting for approximately 20–35% of all salivary gland malignancies [1, 2]. Its annual incidence in Europe is approximately 2–3/1,000,000 [2, 3]. AdCC arises in the major salivary glands and more often in the minor salivary glands of the lip, oral cavity, oropharynx, nasopharynx, nasal cavity, paranasal sinus, larynx and tracheobronchial tree. Occasionally it is seen in the lacrimal and ceruminous glands [1, 4, 5]. AdCC is characterized by slow local progression, extensive perineural spread and a tendency for delayed onset of distant metastasis. Current guidelines consider ^18^F–fluorodeoxyglucose PET/CT (FDG PET/CT) at initial presentation to be of additional value to assess disseminated disease of AdCC and therefore to be of influence on treatment planning in these patients [6]. However, FDG uptake in AdCC is lower than in squamous cell carcinoma (SCC) and not all AdCCs show detectable FDG uptake [7]. Surgery is the primary treatment option, frequently followed by adjuvant radiation therapy to improve local and regional control [1]. Almost half of all patients develop slowly growing distant metastasis within the first five years after diagnosis, mostly to the lungs and skeleton [5, 8]. As a result, long-term mortality is usually caused by distant metastasis or deep local recurrence, of which salvage (re)resection is often impossible [1]. In large European cohort studies, overall five and ten year disease specific survival rates are 68–75% and 52–65%, respectively [8, 9]. Survival is significantly decreased after the diagnosis of distant metastasis, with one and five year survival rates of 54–68% and 7–32%, respectively [8, 10].

The Prostate Specific Membrane Antigen (PSMA), a type II transmembrane glycoprotein of the prostate secretory acinar epithelium, is upregulated in prostate carcinoma and its metastasis [11–13]. Functional imaging of cells expressing PSMA using radiolabelled ligands, e.g. ^68^Gallium-PSMA-HBED-CC (also called ^68^Gallium-PSMA-11) and positron emission tomography combined with computed tomography (commonly referred to as PSMA PET/CT), is primarily used for the detection and (re)staging of prostate cancer [14]. However, prostate specificity of PSMA has been disproved. Clinical experience with PSMA PET/CT for prostate cancer has revealed consistent and significant physiological uptake in normal tissues, including the salivary and lacrimal glands, liver and kidneys [14]. Furthermore, PSMA PET/CT depicts tracer uptake in numerous benign neoplasms and malignancies. Benign lesions include Schwannoma, sarcoidosis, Paget’s disease, desmoid tumours and adenoma of thyroid, pancreas and adrenal gland [15–22]. Nonprostatic malignancies include sarcoma, follicular lymphoma, brain tumours and carcinoma of breast, lung, kidney, thyroid and liver [23–30]. PSMA expression has been frequently investigated by immunohistochemistry and was found to be associated with endothelial cells or tumour neovasculature in malignant disease [31, 32].

PSMA-ligand uptake in metastatic AdCC on PSMA PET/CT has been described previously in 2 case reports [33, 34]. As PSMA targeted tumour-specific treatment will be widely available soon, it might also be suitable in selected cases of AdCC. Data on the presence of PSMA on AdCC are lacking, therefore the aim of this study was to analyse the visualization of local recurrent or distant metastatic AdCC on PSMA PET/CT and to investigate the expression of PSMA on AdCC tumour tissues, both primary and metastatic.

## Methods

### Patient selection

All patients that were diagnosed with AdCC in our institute were retrospectively reviewed. All patients that underwent full-body PSMA PET/CT for the evaluation of AdCC of the head and neck were included. The following clinical parameters were retrieved from the medical files: gender, age, year of diagnosis, tumour location and diameter, presence of local recurrence or distant metastasis.

All performed PSMA PET/CT scans were collected and reviewed by a dedicated board-certified head and neck nuclear medicine physician (B.d.K.) experienced in PSMA PET/CT, in consensus with a head and neck surgeon (R.v.E.). On each scan all areas of focal tracer uptake were assessed. This included both the region of the (former) primary tumour, as well as regional or distant metastasis. Maximum Standardized Uptake Values (SUV_max_) were measured using a freehand isocontour volume of interest in these areas. For reference, SUV_max_ was also measured in normal functioning parotid gland, kidneys and liver. Also, all FDG PET/CTs performed within three months before or after PSMA PET/CT were re-examined for direct comparison.

Representative formaldehyde-fixed, paraffin-embedded tissue blocks of the primary tumours and, when applicable, biopsies or resection specimens of recurrences and/or distant metastases, were retrieved from the pathology archives. Tumour specimens were re-examined by a dedicated head and neck pathologist (S.W.) for the following parameters: type and diameter of the tumour, histopathological grade according to the differentiation of Perzin et al. [35], surgical resection margins and the presence of perineural growth, vaso-invasive growth and bone invasion.

### PET/CT image acquisition

^68^Gallium-HBED-CC was prepared using a GMP-grade ^68^Ge-^68^Ga-generator (GalliaPharm) in combination with an automated system (Modular-Lab Easy), cassettes and buffers as instructed by Eckert & Ziegler Eurotope (Berlin, Germany). 30μg PSMA-HBED-CC (ABX, Radeberg, Germany) was used per preparaton. Labelling quality control was performed by both instant thin layer chromatography and high performance liquid chromatography (Thermo Scientific Dionex UltiMate 3000), in combination with gamma detection. Images were acquired from skull vertex to the thighs using a TruePoint Biograph mCT40 scanner (Siemens, Erlangen, Germany), approximately 60 min after intravenous injection of 2 MBq/kg ^68^Ga-HBED-CC-Glu-NH-CO-NH-Lys(Ahx) or 2 MBq/kg FDG in case of FDG-PET/CT imaging. A low dose CT scan was performed using Care Dose 4D and Care kV, reference parameters: 40 mAs, 120 kV. Subsequently, PET was acquired according to the European Association of Nuclear Medicine (EANM) recommendations with the following parameters: PET with time-of-flight and point spread function (TrueX) reconstruction, 4 iterations, 21 subsets, with a filter of 7.5 mm full width at half maximum [36].

### Immunohistochemistry

Representative paraffin sections 4 μm thick were analysed immunohistochemically using fully automated protocols on the Benchmark XT (Ventana Medical Systems, Tucson, AZ, USA). For the primary antibody, we used a mouse antihuman PSMA monoclonal antibody (3E6; DAKO, Carpinteria, CA) of the IgG1 isotype directed against the internal domain of the PSMA antigen (DAKO, cat. no. M3620, Carpinteria, CA, dilution 1/80). The tissue sections were deparaffinized with xylene and ethanol followed by heat induced epitope retrieval in Ventana Cell Conditioning 1 for 24 min and, subsequently, primary antibody incubation for 60 min. Antigen-antibody reactions were visualized using Ventana OptiViewTM Amplification kit, followed by Ventana OptiViewTM Universal DAB Detection Kit (Optiview HQ Linker 8 min, Optiview HRP Multimer 8 min, Optiview Amplifier H2O2/Amplifier 4 min, Optiview Amplifier Multimer 4 min). Finally, the slides were counterstained with haematoxylin, dehydrated and mounted. PSMA immunohistochemically stained slides were scored for percentage of positive tumour cells.

### Statistics

Statistics were performed using IBM SPSS Statistics for Windows, Version 21.0 (Armonk, NY: IBM Corp., 2012). Patient characteristics and outcome measurements are provided as means ± standard deviation and range, or median with interquartile range (IQR) when these data were not distributed normally.

## Results

Since 1990, fifty-six patients were diagnosed with AdCC at our institute, of which thirteen patients are in active follow-up because of local recurrent or distant metastatic disease. Since October 2015, nine of these patients had been referred for restaging by PSMA PET/CT either because of suspected local recurrence or distant metastasis. These patients, four men and five women, had an average age at diagnosis of 51 ± 15 years (range 31–76 years). Local recurrence occurred in six out of nine patients, with a median time span of 2.8 years after first diagnosis (IQR 2.1–7.6 years). Eight patients eventually developed distant metastasis, on average 5.8 ± 4.1 years after first diagnosis (range 0–12.5 years). Clinical and histopathological tumour characteristics are summarized in Table 1.

**Table 1:** Patient characteristics

No.	Gender	Age at diagnosis (yr)	Year of diagnosis	Tumour site	Diameter (cm)	Perzin grade [35]	Growth pattern
1	M	36	2003	nasal cavity	1.8	2	BI
2	M	31	2003	palate	3.8	2	PN/BI
3	F	54	2005	palate	1.6	2	PN
4	F	58	2007	palate	7.0	1	PN/VI/BI
5	F	61	2008	parotid	3.5	2	PN/VI
6	F	59	2008	parotid	3.5	1	PN
7	F	40	2009	parotid	2.0	3	PN/VI
8	M	41	2013	parotid	0.7	2	PN
9	M	76	2015	nasal cavity	3.3	3	PN/BI

*PN* perineural invasion, *VI* vaso-invasive growth, *BI* bone invasion

### PSMA PET/CT

**Fig. 1:**
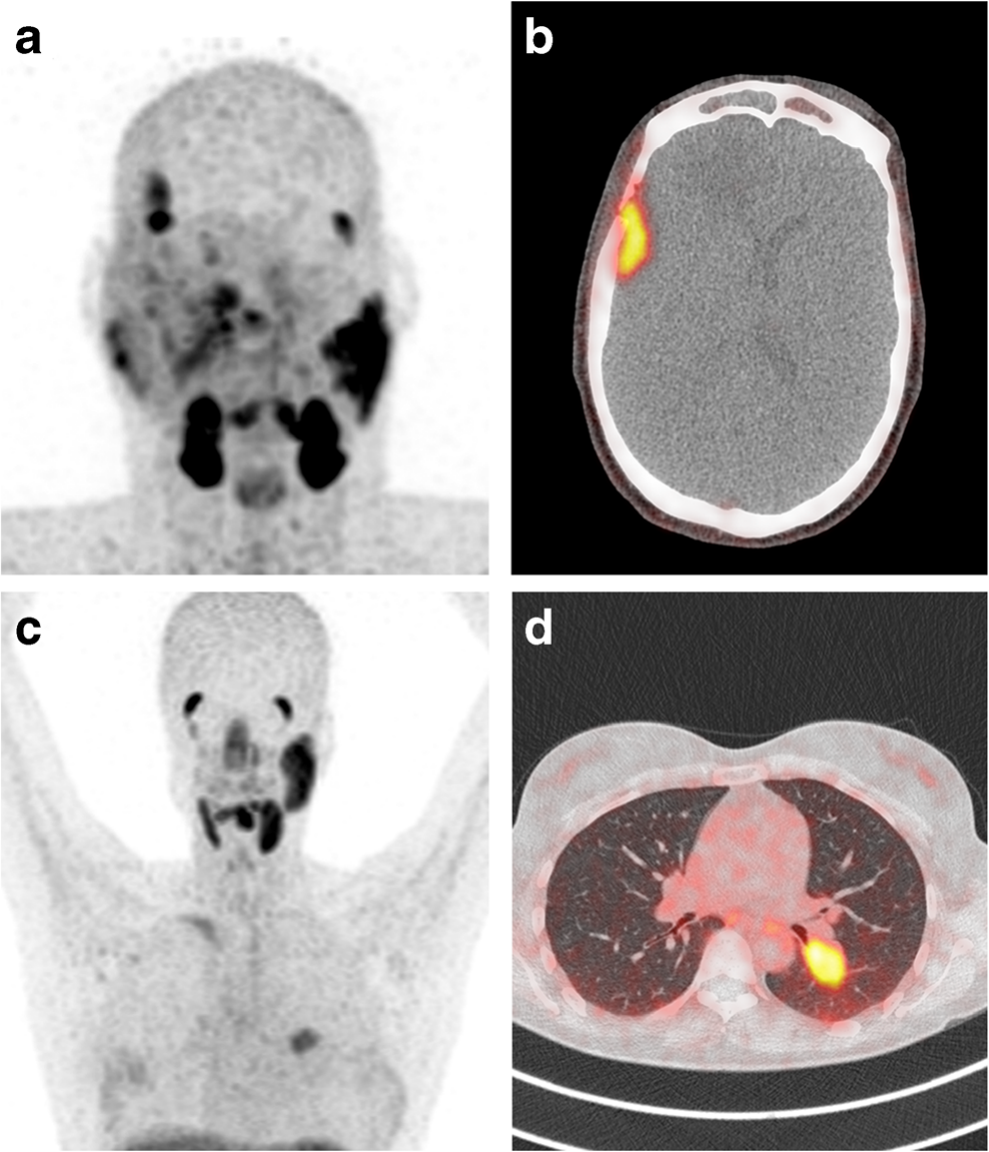
Overview of PSMA PET (**a** and **c**) and axial PSMA PET/CT slides (**b** and **d**) of PSMA-ligand uptake in AdCC. Patient no. 1 (**a** and **b**): nasopharyngeal recurrence and leptomeningeal metastasis; Patient no. 7 (**c** and **d**): local recurrence in the right maxillofacial region and pulmonary metastasis

The mean administered tracer activity was 150 ± 31 MBq (range 107–207 MBq). The time interval between tracer administration and imaging was on average 71 ± 15 min (range 51–91 min). All nine PET/CTs clearly depicted PSMA-ligand uptake in areas of the former primary tumour or localizations of distant metastasis (Table 2). Four PSMA PET/CTs showed tracer accumulation suspected of residual or recurrent AdCC, eight PSMA PET/CTs depicted uptake in areas suspected of distant metastasis. Three scans showed tracer uptake in four new lesions suspected of metastasis; progression of formerly diagnosed metastases was seen in four patients. In one patient, a simultaneous primary prostate carcinoma was detected. When positive, median SUV_max_ in local recurrent AdCC was 2.52 (IQR 2.41–5.95) and median SUV_max_ in distant metastatic AdCC was 4.01 (IQR 2.66–8.71). For reference, median SUVs_max_ in normal parotid, liver and kidneys were 10.94 (IQR 9.01–15.55), 3.83 (IQR 2.99–4.88) and 23.76 (IQR 15.00–35.00), respectively. Three patients underwent concurrent FDG PET/CT within three months from PSMA PET/CT, and data were available for direct comparison. In two patients, PSMA SUV_max_ was comparable to FDG SUV_max_ and in one patient only PSMA PET/CT depicted increased tracer uptake in an area suspected of recurrent AdCC (Table 2). Figure 1 shows projection examples of PSMA-ligand uptake in local recurrent and distant metastatic AdCC.

**Table 2:** PSMA PET/CT, FDG PET/CT and immunohistochemical characteristics

No.	Primary tumour		Local recurrence				Distant metastasis			
	site	IHC (%)	site	PSMA SUV_max_	FDG SUV_max_	IHC (%)	site	PSMA SUV_max_	FDG SUV_max_	IHC (%)
1	2003 nasal cavity	70%	2006 nasopharynx / retrobulbar	*		90%	2016 leptomeningeal	8.71		
			2014 nasopharynx	*		50%				
			2016 nasopharynx / masticator space	7.06		n/a				
2	2003 palate	30%	–				2011 lungs	0	0	70%
							2016 peritoneal	0	0	70%
							2016 liver	0	2.39	
							2016 iliac crest	2.04	2.34	
3	2005 palate	5%	2007 palate	*		30%	2007 lungs	2.66		
							**2016 liver**	4.01		
4	2007 palate	5%	2015 maxilla / retrobulbar	*			2015 intracranial	12.81		
							**2016 vertebra**	3.47		
5	2008 parotid	70%	2015 scalp	2.42		10%	2015 leptomeningeal	2.42		
6	2008 parotid	25%	–				2010 lungs	3.64		
7	2009 parotid	50%	2011 ext. auditory canal	*		30%	2013 lungs	4.68	3.60	
			2015 ext. auditory canal	2.41	4.00	90%				
8	2013 parotid	30%	**2016 parotid**	2.62	1.90**		–			
9	2015 nasal cavity	90%	–				**2015 iliac crest**	12.97		5%
							**2015 lungs**	6.66		

Entries in bold are newly discovered on PSMA PET/CT

IHC: immunohistochemistry; SUV_max_: Maximum Standardized Uptake Value

n/a: biopsy specimen contained no tumour tissue;

* no PET imaging at time of diagnosis of recurrence;** physiological uptake

### PSMA expression by immunohistochemistry

**Fig. 2:**
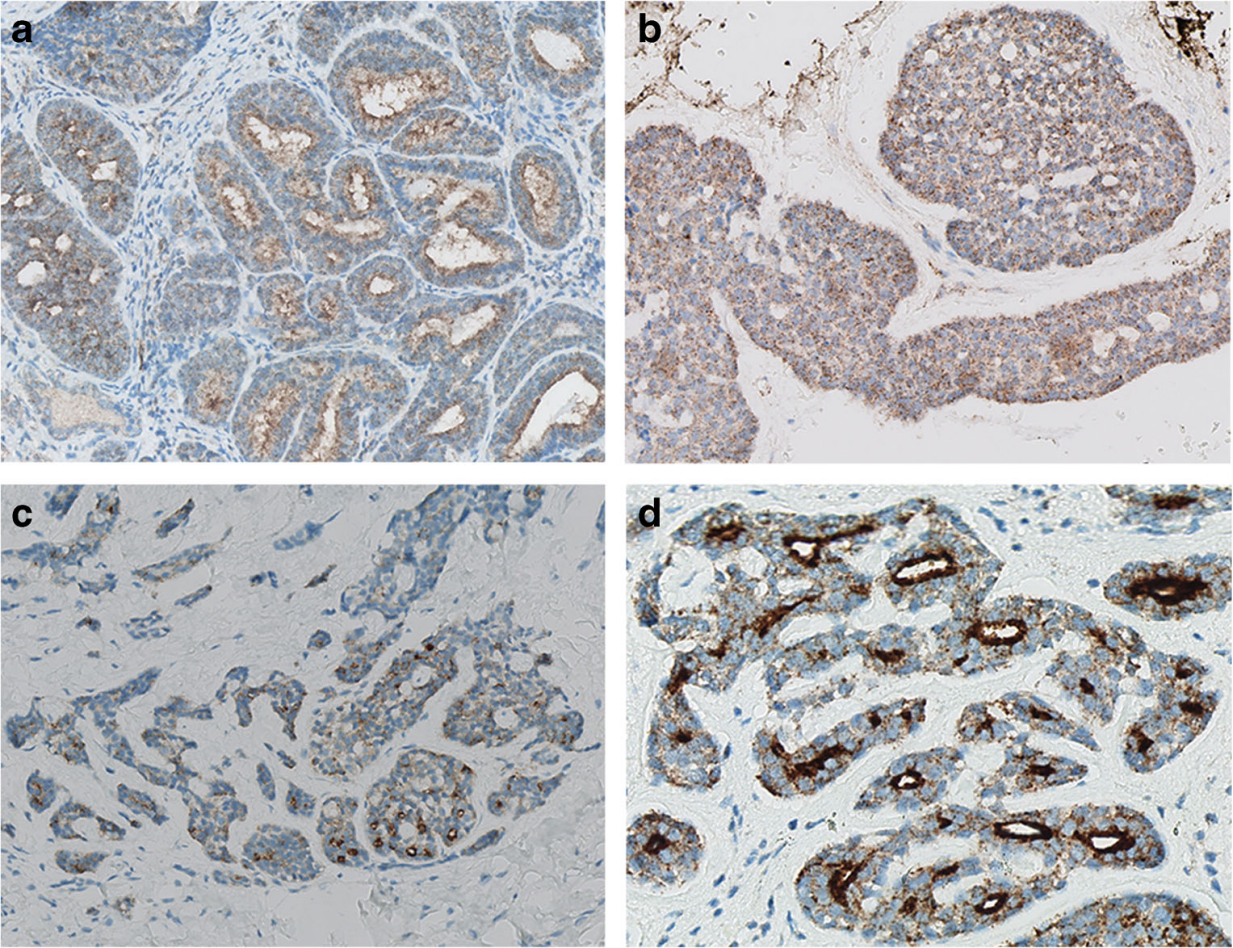
Immunohistochemical PSMA expression in AdCC. *Magnification: a b c 400×; d 700×*. (**a**) nasal cavity primary AdCC, 70%; (**b**) nasopharyngeal recurrence, 90%; (**c**) peritoneal metastasis, 70%; and (**d**) luminal staining of primary parotideal AdCC, 70%

Revision of the nine tumour specimens revealed positive resection margins in all cases of primary resection, for which all these patients received postoperative radiotherapy. Eight tumours demonstrated perineural growth. All primary tumours, as well as all available tumour specimens of local recurrence and distant metastases were positive on PSMA immunohistochemistry. Expression was seen in a granular fashion, mainly cytoplasmic or concentrated at the luminal side of the cell membrane and varied widely between 5 to 90%. Of the primary tumour specimens, a median of 30% of the tumour cells (IQR 15–70%) demonstrated PSMA expression. Examples of different staining patterns are shown in Fig. 2, results per patient are summarized in Table 2.

### Implications for treatment

In four patients, the newly obtained results by PSMA PET/CT led to alteration of treatment. Patient no. 3 is currently undergoing palliative targeted radionuclide therapy with 177-Lutetium-PSMA-617, because of progressive dyspnoea due to pulmonary metastases. Patients no. 7 and 9 were referred for adjuvant irradiation therapy and patient no. 8 was referred to another hospital for experimental chemotherapy. The other five patients received best supportive care only.

## Discussion

This is the first study on a series of patients in which the presence of PSMA in AdCC of the head and neck is demonstrated. PSMA PET/CT was able to visualize local recurrent and/or distant metastatic AdCC in all cases. Furthermore, all primary resection specimens showed PSMA expression on immunohistochemistry, as well as all available tumour tissues of local recurrence and distant metastases.

### PSMA expression

Although little is known about the presence and function of PSMA in salivary gland tissue, the presence of PSMA mRNA in salivary gland extracts had already been detected in 1994 using Western blot analysis [37, 38]. Later, PSMA expression was described in human salivary gland specimen by demonstrating cytoplasmatic immunohistochemical staining of the epithelium of acinar glandular cells [39]. In accordance with two recent case reports on PSMA in AdCC, the present results of functional imaging and immunohistochemistry of primary, local recurrent and distant metastatic AdCC also clearly demonstrate PSMA expression in these tumours [33, 34]. Different protein expression between the primary tumour and residual or local recurrent disease is probably due to upregulation or downregulation of PSMA. However, this tumour heterogeneity seems relatively limited, as all except one (patient no. 2) matched cases show concordant positive expression between primary and recurrent cases.

### PSMA PET/CT vs. FDG PET/CT

Today, ^18^F–fluorodeoxyglucose PET/CT (FDG PET/CT) plays a major role in detection and staging of patients with head and neck cancer, with most studies focusing on SCC [40]. However, it is known that AdCC has different biological characteristics and its FDG-uptake is lower as compared to SCC [7]. A recent study comparing FDG PET/CT and conventional contrast-enhanced CT in patients with AdCC showed similar sensitivity for primary lesion detection. However, in two of the 40 patients the primary tumour showed no FDG uptake at all. FDG PET/CT was superior in identification of lymph node and distant metastasis when compared to conventional CT [7].

In the present study, three patients received concurrent FDG PET/CT and PSMA PET/CT (Table 2). **Patient no. 2** had histopathologically confirmed pulmonary and peritoneal metastases and was suspected of liver metastasis, of which none depicted PSMA-ligand uptake on PSMA PET/CT. Pulmonary nodules and peritoneal metastases showed no FDG uptake either. Although the liver lesion was previously suspected of metastasis on magnetic resonance imaging (MRI), it showed only slightly increased and diffuse FDG uptake just above normal liver parenchyma on FDG PET/CT. FDG and PSMA-ligand uptake in iliac crest metastasis was comparable on both scans.

The area of local recurrence and pulmonary metastases of **patient no. 7** showed similar pathological accumulation of both tracers. **In patient no. 8**, the local recurrence of parotideal AdCC showed only physiological background FDG uptake on FDG PET/CT in the area of the former tumour, in contrast to increased PSMA-ligand uptake suggesting recurrent disease.

Anticipating the presence of high PSMA-ligand uptake in normal salivary gland tissue and the SUV of presently described locally recurrent AdCC, PSMA PET/CT is probably not useful in detecting primary AdCC of major salivary glands. At initial presentation of AdCC, the value of PSMA PET/CT therefore appears comparable to FDG PET/CT, with a main focus on the visualization of nodal or distant metastasis. During follow-up, this study indicates PSMA PET/CT to be able to visualize disease progression, recurrence and distant metastases. PSMA PET/CT is considered to be as reliable as FDG PET/CT, when background tracer accumulation is taken into account.

### PSMA histopathology vs. imaging

In three patients (no. 1, 2 and 4), results of both imaging and immunohistochemistry did not match completely (Table 2). In **patient no. 2**, both primary and metastatic tissues stained positive on PSMA immunohistochemistry. However, there was no uptake of PSMA-ligand in any of the pulmonary, liver or peritoneal metastases on PET/CT. In comparison, there was also no FDG uptake in the pulmonary and peritoneal metastases, and only slight uptake of FDG in the liver metastasis. The absence of metastatic tracer uptake on PSMA PET/CT is due to surgical resection of the pulmonary metastases by a lobectomy in 2011, which made these available for immunohistochemistry. The remaining suspicious pulmonary nodules, which were not cytologically proven malignant, were only 5 mm or less in diameter. If indeed these nodules were metastases, insufficient tracer-uptake in small tumour volumes may be an explanation, analogous to FDG PET/CT [41]. The invisibility of immunohistochemically positive liver and peritoneal metastases on PSMA PET/CT may be explained by the physiological distribution of PSMA in these tissues. We assume the PSMA-ligand uptake in these liver and peritoneal metastases to be equal to or less than background uptake of PSMA [14].

In **patient no. 1**, an early 2016 MRI was suspicious of nasopharyngeal recurrence and leptomeningeal metastasis, which was supported by high tracer uptake on PSMA PET/CT one month later (SUV_max_ 7.06 and 8.71 respectively). Unfortunately, a biopsy specimen of this area contained no tumour tissue and histopathological samples of the leptomeningeal lesions were not obtained. The primary tumour of **patient no. 4** was weakly positive on PSMA immunohistochemistry. The early 2015 local recurrence, diagnosed on previous MRI, depicted no tracer uptake on 2016 PSMA PET/CT, probably because it was previously treated by stereotactic radiotherapy. Based on this PSMA PET/CT, it is therefore not entirely clear whether the reported local abnormalities indicate recurrent disease or should be classified as post-irradiation effects. In addition, there was intracranial and vertebral PSMA-ligand uptake, highly suspicious of distant metastases. Histopathological confirmation of these lesions was not sought due to their location.

### New treatment option

As shown in this series, recurrent and metastatic disease is a relevant issue in AdCC patients. As a result, survival rates have not improved over the past decades, as no progress has been made in developing new treatment modalities. Disease specific survival is mainly affected by stage, margin status and histopathological grade, i.e. a solid growth pattern, and subsequent to these the occurrence of distant metastasis. Postoperative radiotherapy does improve locoregional control, but does not affect survival [8, 10, 42]. In advanced metastatic castration-resistant prostate carcinoma, patients are now receiving a radionuclide tumour-specific treatment directed against PSMA-overexpressing prostate cancer cells. With this therapy, a PSMA-ligand is labelled with the β-emitter lutetium-177 which causes internal radiation [43]. The results are encouraging with a ≥ 50% decrease of plasma prostate-specific antigen (PSA) in 45% of patients after one cycle of ^177^Lu-PSMA-617, as well as a decrease of SUV_max_ on PET/CT [43, 44]. Treatment with this radionuclide was deemed to be safe and well tolerated in a large multicenter retrospective study. Bone-marrow depression, renal failure and xerostomia are reported side-effects [44]. In comparison with PSMA PET/CT imaging of prostate cancer, using the same radiotracer, prostate cancer tumour lesions have an average SUV_max_ of 13.3 ± 14.6 (range 0.7–122.5) [45]. Uptake of PSMA-tracer in AdCC, given our results, is considered moderate. Therefore, we are currently evaluating whether this ^177^Lu-PSMA-617-therapy, using the same therapy regimen and activity which has already shown to be associated with limited adverse effects in prostate cancer, will be as effective in patients suffering from irresectable recurrent and/or metastatic AdCC. In most malignancies, other than prostate cancer, PSMA is localized in the neovasculature [23, 27, 31, 32, 46]. Because the localization of PSMA in AdCC is mainly cytoplasmic or concentrated at the luminal side of the cell membrane, therapy with Alpha-emitting ^225^Ac-PSMA-617 might also be a viable therapeutic option [47].

This study has some limitations. As this was a retrospective analysis, in five recurrent lesions of four patients PSMA PET/CT was not available at the time of diagnosis. However, only two recurrent cases had no PSMA PET imaging at all, and moreover all PSMA PET/CTs showed concordant positive tracer uptake. More importantly, only two patients suspected of recurrence underwent concurrent FDG PET/CT, of which one was suggested to have disease recurrence and one was underestimated as physiological uptake. In detection of distant sites, eight patients with eleven sites were detected by PSMA PET/CT, of which two could be compared to FDG PET/CT and showed similar tracer accumulation. The results of this study should be interpreted as a preliminary descriptive analysis of PSMA in these tumours. The additive value of PSMA PET/CT over FDG PET/CT needs to be further investigated.

## Conclusion

PSMA PET/CT is able to detect and visualize local recurrent and distant metastatic AdCC. Additionally, PSMA-specific targeting is supported by high PSMA expression on immunohistochemistry. When compared to FDG PET/CT, we found concordant tracer uptake without underdiagnosing clinically relevant disease progression of both local recurrence and distant metastasis.
